# Cardiac involvement in cystic fibrosis evaluated using cardiopulmonary magnetic resonance

**DOI:** 10.1007/s10554-021-02496-6

**Published:** 2022-01-07

**Authors:** Jakub Lagan, Josephine H. Naish, Joshua Bradley, Christien Fortune, Charlie Palmer, David Clark, Erik B. Schelbert, Matthias Schmitt, Rowland Bright-Thomas, Christopher A. Miller

**Affiliations:** 1 Manchester University NHS Foundation Trust, Wythenshawe Hospital, Southmoor Road, Wythenshawe, Manchester, M23 9LT England, UK; 2 Division of Cardiovascular Sciences, School of Medical Sciences, Faculty of Biology, Medicine and Health, Manchester Academic Health Science Centre, University of Manchester, Oxford Road, Manchester, M13 9PL England, UK; 3 Department of Medicine, University of Pittsburgh School of Medicine, Pittsburgh, PA USA; 4 UPMC Cardiovascular Magnetic Resonance Center, Heart and Vascular Institute, Pittsburgh, PA USA; 5 Clinical and Translational Science Institute, University of Pittsburgh, Pittsburgh, PA USA; 6 Wellcome Centre for Cell-Matrix Research, Division of Cell-Matrix Biology & Regenerative Medicine, School of Biology, Faculty of Biology, Medicine & Health, Manchester Academic Health Science Centre, University of Manchester, Oxford Road, Manchester, M13 9PT England, UK

**Keywords:** Cystic fibrosis, Cardiac magnetic resonance, Myocardial fibrosis, Myocardial inflammation, Parametric mapping

## Abstract

Cystic fibrosis (CF) transmembrane conductance regulator is expressed in myocardium, but cardiac involvement in CF remains poorly understood. The recent development of a combined cardiopulmonary magnetic resonance imaging technology allows for a simultaneous interrogation of cardiac and pulmonary structure and function. The aim of this study was to investigate myocardial manifestations in adults with CF, both in a stable state and during an acute respiratory exacerbation, and to investigate the relationship between cardiac and pulmonary disease. Healthy adult volunteers (n = 12) and adults with CF (n = 10) were studied using a multiparametric cardiopulmonary magnetic resonance protocol. CF patients were scanned during an acute respiratory exacerbation and re-scanned when stable. Stable CF was associated with left ventricular dilatation and hypertrophy (LVH; left ventricular mass: CF 59 ± 9 g/m^2^ vs. control 50 ± 8 g/m^2^; p = 0.028). LVH was predominantly driven by extracellular myocardial matrix expansion (extracellular matrix mass: CF 27.5 ± 3.4 g vs. control 23.6 ± 5.2 g; p = 0.006; extracellular volume [ECV]: CF 27.6 [24.7–29.8]% vs. control 24.8 [22.9–26.0]%; p = 0.030). Acute CF was associated with an acute reduction in left ventricular function (ejection fraction: acute 57 ± 3% vs. stable 61 ± 5%; p = 0.025) and there was a suggestion of myocardial oedema. Myocardial oedema severity was strongly associated with the severity of airflow limitation (r = − 0.726, p = 0.017). Multiparametric cardiopulmonary magnetic resonance technology allows for a simultaneous interrogation of cardiac and pulmonary structure and function. Stable CF is associated with adverse myocardial remodelling, including left ventricular systolic dilatation and hypertrophy, driven by myocardial fibrosis. CF exacerbation is associated with acute myocardial contractile dysfunction. There is also a suggestion of myocardial oedema in the acute period which is related to pulmonary disease severity.

**Supplementary Information:**

The online version contains supplementary material available at 10.1007/s10554-021-02496-6.

## Introduction

Cystic fibrosis (CF), the most common lethal autosomal recessive disorder in Caucasian populations, is a multisystem condition caused by mutations in the CF transmembrane conductance regulator (CFTR) gene [1]. The resulting impaired transport of chloride and other ions leads to hyperviscous secretions. Pulmonary manifestations remain the main cause of morbidity and mortality and forced expiratory volume in one second (FEV_1_) is the strongest indicator of prognosis [1].

CFTR is expressed in myocardium [2], but while cor pulmonale is well recognised, the heart is not generally considered part of the primary disease expression. Existing data regarding cardiac manifestations are conflicting and it is unclear whether cardiac abnormalities, if present, are related to disease severity [3–5]. With improving life expectancy, understanding cardiac involvement in CF is important in terms of quality of life, prognosis and transplant candidacy [6].

Magnetic resonance imaging (MRI) can provide unique in vivo insight into myocardial and lung injury [7]. Myocardial cine imaging allows for cardiac functional analysis [8]. Late gadolinium enhancement imaging (LGE) has been extensively validated in detecting focal replacement myocardial fibrosis [9]. Myocardial parametric mapping, including T1, T2 and extracellular volume (ECV) mapping, provides a robust assessment of tissue composition, specifically a free water content (i.e. oedema) and the degree of extracellular collagen deposition (i.e. focal and diffuse fibrosis) [10]. ECV is a well validated tool to measure the extracellular matrix fraction and as such can be used to differentiate the cellular from extracellular left ventricular mass [10]. Pulmonary T1 mapping was shown to detect pulmonary inflammation and pulmonary tissue destruction and fibrosis [11–13]. Dynamic contrast enhanced MRI (DCE-MRI) with contrast agent kinetics modelling allows for the assessment of pulmonary perfusion and capillary permeability (K^trans^; transfer constant), a surrogate marker of inflammation [14–16]*.*

This study aimed to investigate myocardial manifestations of CF, during the acute exacerbation and in the stable state of the disease, and to analyse the relationship between cardiac and pulmonary disease in CF using a combined cardiopulmonary MRI technology.

## Material and methods

### Study design

This prospective research study aimed to apply a combined cardiopulmonary MRI protocol in healthy volunteers and, for the first time, in patients with CF during an acute pulmonary exacerbation and in a stable period in order to investigate the relationship between cardiac and pulmonary disease.

### Study population

Consecutive consenting adult patients with genetically confirmed CF admitted to Manchester Adult CF Centre, Manchester University NHS Foundation Trust with acute respiratory exacerbation were prospectively recruited [17]. Exclusion criteria included: any history of cardiac disease, contraindication to MRI, glomerular filtration rate < 40 ml/min/1.73m^2^. Healthy volunteers with no cardiovascular symptoms, no history of medical conditions and normal electrocardiogram (ECG) were prospectively recruited to act as controls.

### Study procedures

Healthy volunteers underwent blood sampling (blood count, renal function, c-reactive protein (CRP), high sensitivity troponin I (hsTnI)), ECG and MRI.

CF patients underwent identical blood sampling, ECG and MRI and additional measurement of spirometry (EasyOne portable spirometer; NDD Medical technologies, Zurich, Switzerland) and blood gas analysis during the acute hospital admission (acute CF). Investigations were repeated when patients were deemed clinically stable, a minimum of 5 weeks following the acute admission (stable CF). Findings were compared with the healthy volunteers.

### Combined cardiopulmonary MRI

MRI was performed at 1.5 T scanner (Avanto, Siemens Medical Imaging). Scan duration was 60 min. The MRI protocol is described in the supplementary material and shown in Supplementary Fig. 1. It was also previously described in detail [7]. In brief, it included: myocardial steady-state free precession cine imaging, myocardial and pulmonary native T1 mapping, myocardial T2 mapping, myocardial and pulmonary DCE-MRI, post contrast myocardial T1 mapping and LGE imaging.

### MRI analysis

MRI analysis (performed by JL and CAM; both holding level 3 cardiovascular MRI accreditation; Society for Cardiovascular Magnetic Resonance) is described in the supplementary material and was also described previously in detail [7]. In brief, cardiac volumetric analysis was performed using Circle CVI42 (Circle Cardiovascular Imaging, Canada) according to guidelines [8]. Parametric maps were analysed in Horos (Horos2K v2.2.0 The Horos Project). Myocardial ECV was calculated using same-day haematocrit as described previously [18]. LV extracellular matrix mass (g) was calculated by multiplying LV mass by ECV [10]. LV cellular mass (g) was calculated by multiplying LV mass by (100%-ECV). DCE imaging was analysed using a custom written Matlab code (v9.0, The MathWorks, USA). Contrast agent kinetics were modelled using an extended version of a Kety model to calculate myocardial and pulmonary capillary permeability (K^trans^) and pulmonary extracellular volume fraction (Ve) [14]. Pulmonary blood flow (F) was calculated by deconvolution of the first pass dynamic data as described previously [14, 15].

### Statistical analysis

This was the first study to apply cardiac MRI in CF thus there were no data to base a power calculation upon. Using data from other populations, 10 CF patients were required to detect an absolute minimum difference, between acute and stable scans, of 2% in terms of absolute change in ECV, with 80% power at a 5% significance level (2-sided), assuming a standard deviation of within-patient differences equal to 2% [18].

Data are summarised using mean and standard deviation or median and interquartile range, and compared using t tests or non-parametric equivalents, as appropriate. Correlation analysis was performed using Pearson or Spearman correlation. P value of ≤ 0.05 was considered significant. Analyses were performed using SPSS (v22, IBM).

## Results

### Participants characteristics

Ten CF patients with an acute respiratory exacerbation were recruited; median age 28 (25–36) years; 2 (20%) female. Baseline characteristics are summarised in Table 1. Mean admission arterial partial pressure of oxygen was 8.9 ± 0.9 kPa and FEV_1_ was 1.4 ± 0.4L/s. Details of antibiotic treatment are provided in Supplementary Material. Median interval from admission to acute MRI scan was 12 (8–16) days. No patient had detectable hsTnI. Twelve healthy volunteers were recruited as controls; median age 37 (27–45) years; 6 (50%) female; Table 1.

**Table 1:** Patient characteristics

Parameter	Control (n = 12)	Acute CF (n = 10)	p value (vs. control)	Stable CF (n = 9)	p value (vs. control)	p value (acute vs. stable CF)
Demographics						
Age (years)	37 (27–45)	28 (25–36)	0.129			
Gender (female)	6 (50%)	2 (20%)	0.204			
BSA (m^2^)	1.9 (1.7–2.2)	1.8 (1.7–1.9)	0.429	1.8 (1.7–1.8)	0.434	
Weight	74 (59–93)	65 (63–73)	0.355	65 (63–69)	0.374	
CF manifestations						
Pancreatic insufficiency		10 (100%)				
DM		6 (60%)				
PA colonisation		7 (70%)				
Biliary disease		4 (40%)				
ABPA		2 (20%)				
Laboratory findings						
CRP (mg/l)	1 (0–2)	32 (14–121)	0.001	19 (7–24)	0.001	0.058
WBC (× 10^9^/l)	5.9 (4.9–7.0)	12.0 (10.0–15.8)	0.001	10.3 (8.6–12.4)	0.001	0.066
ECG findings						
PR duration (ms)	140 (120–170)	140 (140–165)	0.502	160 (130–170)	0.556	0.705
QRS duration (ms)	80 (80–90)	80 (80–85)	0.633	80 (80–100)	0.915	0.317
QTc (ms)	394 ± 21	413 ± 23	0.079	414 ± 23	0.063	0.750
Spirometry						
FEV_1_ (l/s)		1.4 ± 0.4		1.7 ± 0.5		0.028
FEV_1_%		34.0 ± 10.4		41.2 ± 11.8		0.025
FVC (l)		2.6 ± 0.6		3.1 ± 0.4		0.026
FVC%		51.6 ± 10.8		62.4 ± 5.3		0.017

Data presented as mean ± standard deviation or median (interquartile range) depending on distribution

*ABPA* allergic bronchopulmonary aspergillosis, *BSA* body surface area, *CF* cystic fibrosis, *CRP* c reactive protein, *DM* diabetes mellitus, *FEV*_*1*_ forced expiratory volume in 1 s, *FEV*_*1*_*%* percentage of predicted forced expiratory volume in 1 s, *FVC* forced vital capacity, *FVC%* percentage of predicted forced vital capacity, *WBC* white blood cell

Nine CF patients underwent MRI in the stable stage (one became too unwell to re-attend). Median time from admission to stable MRI was 85 (52–398) days. Mean FEV_1_ was 1.7 ± 0.5 L/s. Five patients had severe disease (percentage of predicted FEV_1_ (FEV_1_%) < 40%) and 5 had moderate disease (FEV_1_% 40%–69%). All spirometry indices improved between acute and stable assessments. CRP and white cell levels improved although these remained elevated compared to controls.

### Myocardial manifestations of CF

**Fig. 1:**
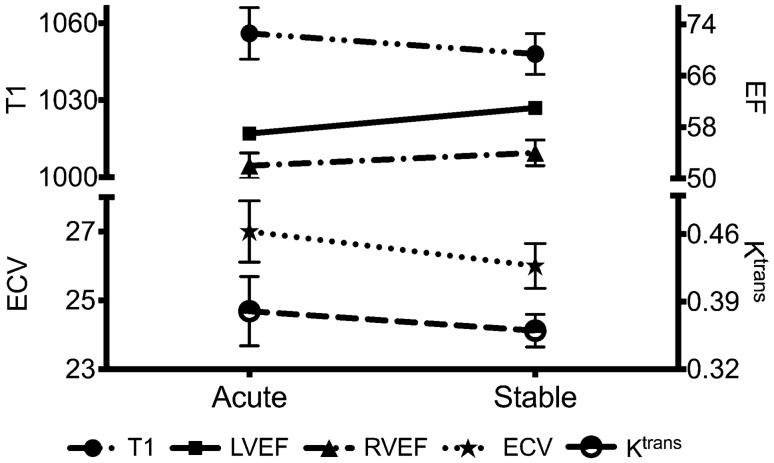
Changes in cardiac structure and function between acute respiratory exacerbation and stable period. There was a reduction in myocardial capillary permeability (K^trans^; min^−1^) between acute respiratory exacerbation and stable stage. Likely as a result, myocardial extracellular volume (ECV; %) and T1 relaxation (ms) time both reduced, in keeping with a reduction in myocardial oedema. In turn, left and right ventricular contractile function improved (*LVEF* left ventricular ejection fraction; %; *RVEF* right ventricular ejection fraction; %)

Acute CF exacerbation was associated with a significant reduction in left ventricular ejection fraction (LVEF), mediated by an increase in LV end systolic volume (control LVEF 63 ± 5% vs. acute CF LVEF 57 ± 3%, p = 0.004; Table 2; Fig. 1). LVEF improved significantly in the stable period (stable CF LVEF 61 ± 5%; p = 0.025 compared to acute CF) such that it was no different to that in controls (p = 0.27). Acute CF exacerbation was also associated with a significant reduction in right ventricle ejection fraction (RVEF), mediated by an increase in RV end systolic volume. RVEF improved in the stable stage but remained borderline reduced in comparison to controls (p = 0.053).

**Table 2:** Cardiac MRI measurements

Parameter	Control (n = 12)	Acute CF (n = 10)	p value (vs. control)	Stable CF (n = 9)	p value (vs. control)	p value (acute vs. stable CF)
Left ventricle						
LV EDV/BSA (ml/m^2^)	81 ± 13	91 ± 14	0.112	92 ± 10	0.052	0.730
LV ESV/BSA (ml/m^2^)	30 ± 6	39 ± 7	0.003	36 ± 5	0.018	0.089
LV EF (%)	63 ± 5	57 ± 3	0.004	61 ± 5	0.270	0.025
LV mass/BSA (g/m^2^)	50 ± 8	58 ± 7	0.016	59 ± 9	0.028	0.885
Right ventricle						
RV EDV/BSA (ml/m^2^)	84 ± 12	92 ± 14	0.169	94 ± 11	0.067	0.614
RV ESV/BSA (ml/m^2^)	34 ± 6	45 ± 10	0.007	43 ± 78	0.007	0.842
RV EF (%)	59 ± 6	51 ± 7	0.006	54 ± 6	0.053	0.230
Atria						
LA area/BSA (cm^2^/m^2^)	12 ± 1	12 ± 2	0.929	11 ± 1	0.315	0.048
RA area/BSA (cm^2^/m^2^)	12 ± 2	10 ± 1	0.008	10 ± 1	0.014	0.607
Myocardial tissue characterisation						
LGE (g)	0 (0–0)	0.92 (0.53–1.35)	< 0.001	1.04 (0.79–1.09)	< 0.001	0.953
LGE (% LV mass)	0 (0–0)	0.92 (0.42–1.39)	< 0.001	0.91 (0.75–1.29)	< 0.001	0.953
T1 (ms)	1021 ± 25	1056 ± 31	0.008	1048 ± 24	0.027	0.246
T2 (ms)	48 ± 2	49 ± 2	0.697	48 ± 2	0.666	0.344
ECV (%)	24.8 (22.9–26.0)	27.6 (24.7–29.8)	0.030	26.2 (25.4–27.2)	0.047	0.515
Extracellular matrix mass (g)	23.6 ± 5.2	28.7 ± 4.9	0.027	27.5 ± 3.41	0.006	0.571
Cellular mass (g)	72.6 ± 18.5	76.7 ± 11.5	0.56	77.83 ± 13.8	0.49	0.62
K^trans^ (min^−1^)	0.33 ± 0.05*	0.39 ± 0.11	0.298	0.37 ± 0.05	0.192	0.617

Data presented as mean ± standard deviation or median (interquartile range) depending on distribution. *n = 5

*ECV* extracellular volume fraction, *EDV* end diastolic volume, *EF* ejection fraction, *ESV* end systolic volume, *K*^*trans*^ transfer constant, *LA* left atrium, *LGE* late gadolinium enhancement, *LV* left ventricle, *RA* right atrium, *RV* right ventricle. Other abbreviations as per Table 1

**Fig. 2:**
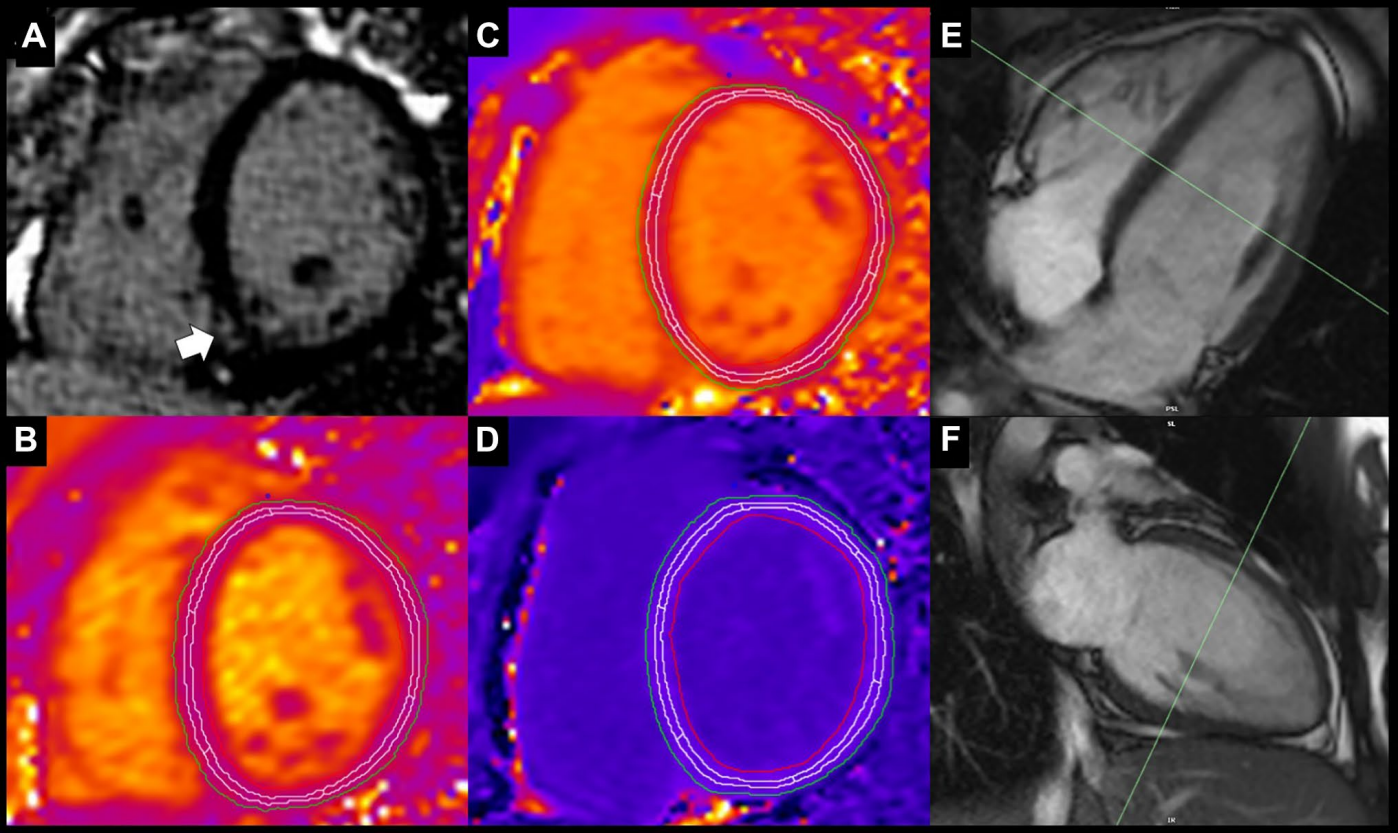
Myocardial tissue characterisation in cystic fibrosis. Representative endocardial and epicardial regions of interest for T1 and T2 mapping are shown. Mid third of the left ventricle (LV) wall was used for analysis. **A** Inferior right ventricle insertion point late enhancement (arrow). **B** Mid LV T2 mapping (T2 = 46 ms). **C** Mid LV native T1 mapping (T1 = 1088 ms). **D** Mid LV post contrast T1 mapping (post contrast T1 = 588 ms). **E, F** Long axis cine imaging showing the position of mid LV short axis parametric mapping slices

Acute CF exacerbation was associated with a significant increase in myocardial ECV (control 24.8% (22.9–26.0%) vs. acute CF 27.6% (24.7–29.8%), p = 0.030) and T1 (control 1021 ± 25 ms vs. acute CF 1056 ± 31 ms, p = 0.008). Myocardial ECV and T1 both improved in the stable period of the disease (ECV 26.2% (25.4–27.2%); T1 1048 ± 24 ms) but remained higher than in controls. There was no difference in T2 values. Nine (90%) CF patients exhibited non-ischaemic RV insertion point LGE. There were no other focal LGE patterns observed and there was no evidence of previous myocardial infarction in any of the patients. Capillary permeability (K^trans^) was non-significantly higher in acute CF compared to controls (Table 2). Figure 2 shows an example of myocardial tissue characterisation sequences.

Stable CF was associated with LV hypertrophy (LVH; LV mass: control 50 ± 8 g/m^2^ vs. stable CF 59 ± 9 g/m^2^; p = 0.028), which was predominantly driven by increased extracellular matrix mass (control 23.6 ± 5.2 g vs. stable CF 27.5 ± 3.41 g; p = 0.006). Stable CF was also associated with LV dilatation (LV end systolic volume: control 30 ± 6 ml/m^2^ vs. stable CF 36 ± 5 ml/m^2^; p = 0.018; LV end diastolic volume: control 81 ± 13 ml/m^2^ vs. stable CF 92 ± 10 ml/m^2^; p = 0.052) and RV dilatation (Table 2).

QTc interval was trending to be longer in both acute and stable CF, compared to controls (control 394 ± 21 ms vs. acute CF 413 ± 23 ms; p = 0.079; vs stable CF 414 ± 23 ms; p = 0.063).

### Pulmonary MRI indices

**Fig. 3:**
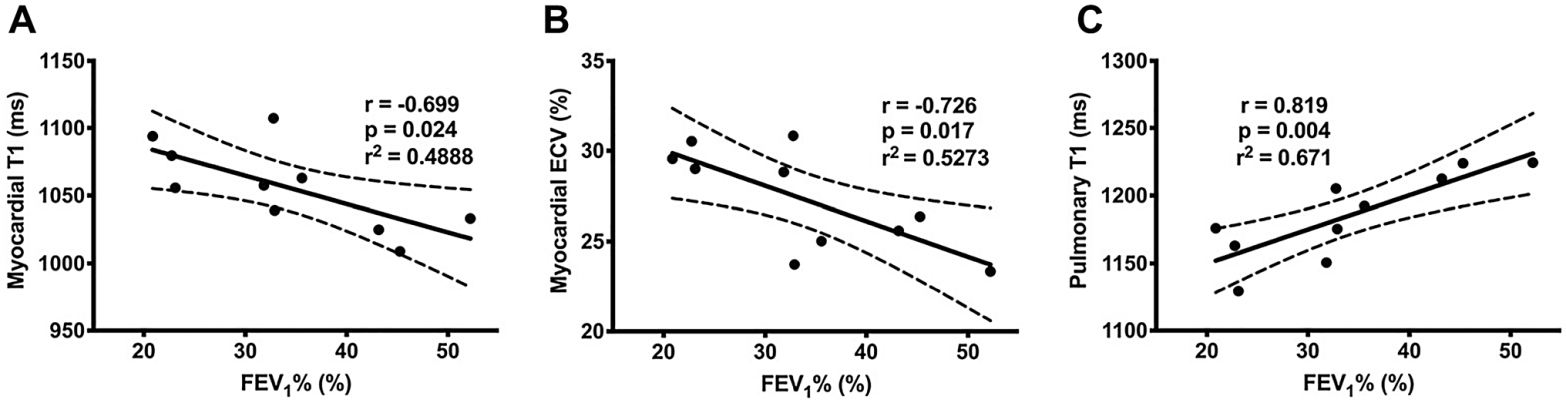
Relationship between myocardial and pulmonary tissue characteristics and percentage of predicted normal forced expiratory volume in one second (FEV_1_%). In acutely exacerbating cystic fibrosis patients, **a** myocardial T1 relaxation time and **b** myocardial extracellular volume (ECV), both indicative of myocardial oedema, showed strong negative correlations with FEV_1_%. **c** Pulmonary T1 relaxation time was strongly correlated with FEV_1_%

**Fig. 4:**
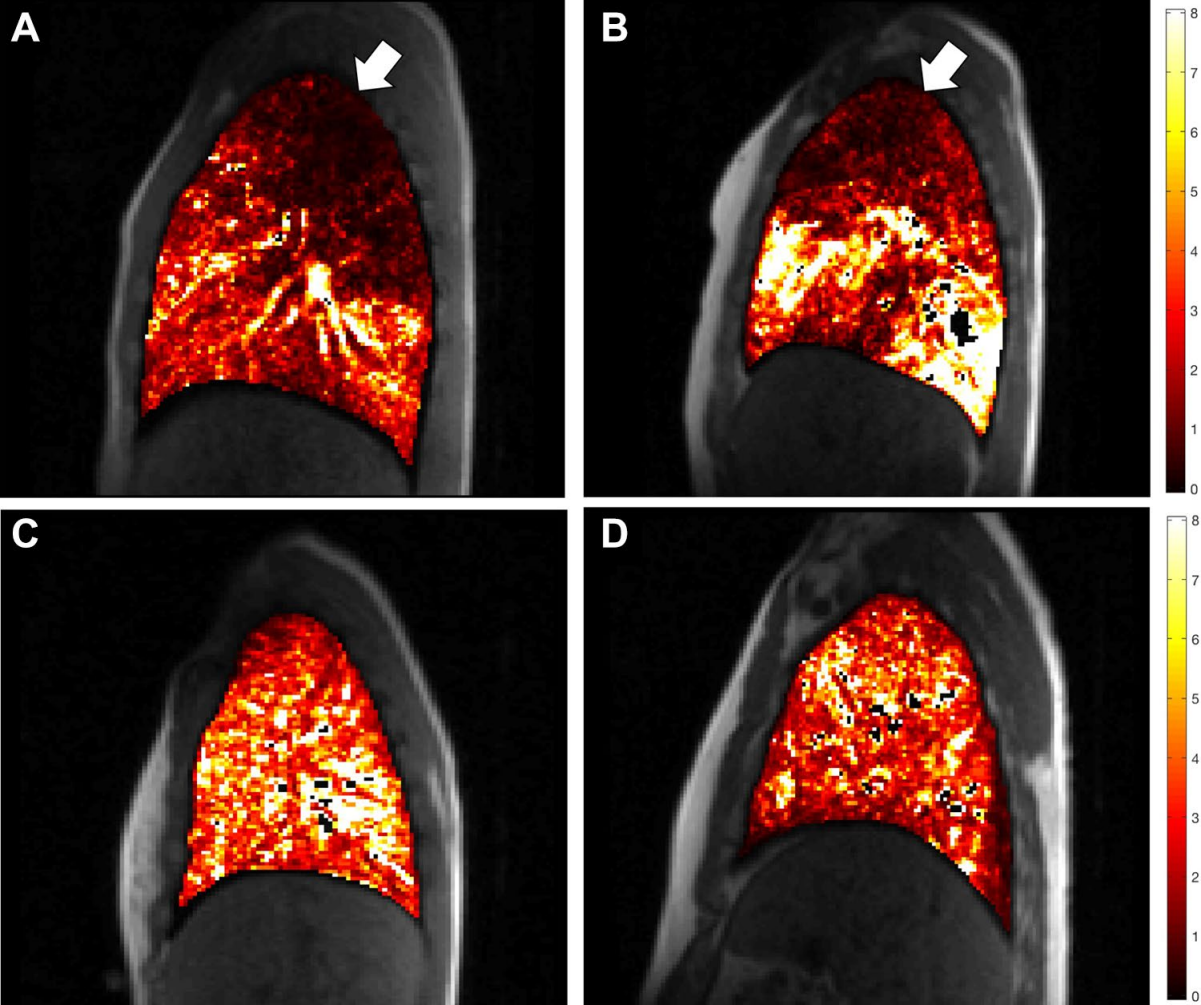
Pulmonary tissue blood flow maps. **a, b** Right lung in patients with cystic fibrosis. There is a visible reduction in pulmonary tissue blood flow in right upper lobes (arrows). **c, d** Right lung in healthy controls

Both acute and stable CF were associated with significantly reduced pulmonary T1 (control 1266 ± 42 ms vs. stable CF 1198 ± 28 ms; p = 0.003 and vs. acute CF 1185 ± 32; p = 0.001; Table 3). Pulmonary T1 correlated strongly with FEV_1_% (r = 0.819, p = 0.004; Fig. 3). CF was also associated with significantly reduced pulmonary tissue blood flow, which was most pronounced in the upper lobes (Table 3; Fig. 4). There was a non-significant increase in pulmonary tissue blood flow during acute exacerbation, although it remained lower than in controls.

**Table 3:** Pulmonary MRI measurements

Parameter	Controls	Acute CF (n = 10)	p value (vs. control)	Stable CF (n = 9)	p value (vs. control)	p value (acute vs. stable CF)
Pulmonary tissue characterisation						
T1 (ms)	1266 ± 42^†^	1185 ± 32	0.001	1198 ± 28	0.003	0.643
Ve (%)	24.5 (14.1–29.4)*	26.4 (21.0–29.5)	0.462	24.1 (21.8–29.4)	0.641	0.767
K^trans^ (min^−1^)	0.27 ± 0.07*	0.23 ± 0.07	0.399	0.26 ± 0.07	0.791	0.445
Pulmonary tissue blood flow						
Both lungs	2.84 (1.91–3.39)*	1.63 (1.45–1.89)	0.027	1.51 (1.02–1.71)	0.053	0.594
Right upper lobe	2.84 (1.71–3.01)*	0.99 (0.73–1.30)	0.007	0.83 (0.57–1.88)	0.014	0.260
Right lower lobe	3.33 (1.99–4.00)*	1.92 (1.61–2.16)	0.066	1.67 (1.27–3.43)	0.125	0.441
Right middle lobe	2.72 (1.82–3.81)*	1.93 (1.34–2.46)	0.142	1.51 (0.94–2.58)	0.072	0.859
Left upper lobe	1.94 (1.67–3.43)*	1.42 (1.15–2.03)	0.053	1.09 (0.78–1.68)	0.028	0.594
Left lower lobe	2.47 (1.93–4.00)*	2.46 (1.81–2.58)	0.463	1.87 (1.41–2.68)	0.125	0.374

Data presented as mean ± standard deviation or median (interquartile range) depending on distribution. Measurements are averaged for both lungs unless stated otherwise. Pulmonary blood flow units are ml blood/ml tissue/min

^∆^n = 9; ^†^n = 8; *n = 5; Ve—pulmonary extracellular volume fraction. Other abbreviations as per Table 2

### Relationship between cardiac and pulmonary disease in CF

Myocardial ECV (r = − 0.726, p = 0.017) and T1 (r = − 0.699, p = 0.024) showed strong negative correlations with FEV_1_% in acutely exacerbating CF patients (Fig. 3).

## Discussion

Applying a multiparametric cardiopulmonary MRI technique to adults with CF demonstrates that acute respiratory exacerbations of CF are associated with significant acute myocardial contractile dysfunction. There is also a suggestion of myocardial oedema in the acute period which is related to pulmonary disease severity. In the stable state, CF patients demonstrate adverse myocardial remodelling, including systolic LV dilatation and LVH, driven by focal replacement and diffuse interstitial myocardial fibrosis. RV dilatation and dysfunction were also evident despite no clinical evidence of cor pulmonale.

It is increasingly recognised that lung diseases are associated with cardiovascular diseases, independent of shared risk factors, and that, in turn, cardiovascular diseases independently contribute to all-cause mortality in patients with lung conditions [19, 20]. The association is particularly strong for heart failure and is independent of the increased risk of coronary artery disease [20]. The mechanisms underlying these associations are unclear, but myocardial inflammation occurring as part of systemic inflammation, with subsequent interstitial myocardial fibrosis, leading to mechanical, electrical and vasomotor myocardial dysfunction, is a widely held hypothesis [21]. However, evidence for this is limited, in part because existing methods to investigate these disease mechanisms, such as circulating markers of inflammation and fibrosis, are not organ specific.

The cardiopulmonary MRI technology provides direct and simultaneous interrogation of cardiac and pulmonary pathophysiology, and their relationship, including mechanical function, inflammation, fibrosis and blood flow characteristics. Indeed, the myocardial injury identified using the MRI protocol was not evident from circulating biomarkers or ECG. The accuracy and reproducibility of MRI mean that relatively small sample sizes are often sufficient [22]. Echocardiography studies in CF have produced conflicting findings, in part because of the high variability of echocardiographic measurements [3–5]. Ours is the first study to apply cardiac MRI in CF.

Preclinical studies demonstrate that loss of CFTR function has a primary myocardial effect. CFTR is important for regulation of resting cardiomyocyte membrane potential and action potential duration [2]. Acute inhibition of CFTR leads to a transient decrease in cardiomyocyte contractility, increase in intracellular calcium concentration and inhibition of protein kinase A activity [23]. In order to normalise contractility, Ca2^+^/calmodulin-dependent kinase II (CaMKII) and Ca^2+^-activated chloride channels are chronically activated [23]. Whilst this is sufficient to maintain resting contractility, response to β-adrenergic stimulation is impaired [24]. Furthermore, CaMKII upregulation is associated with LV dilatation, LVH and QT prolongation [25, 26]. The LV hypertrophy observed in preclinical models is independent of other organ involvement.

Our findings, which are remarkably consistent with the preclinical findings, provide novel insight into cardiac involvement in CF in humans. Specifically, we demonstrate that in the stable state, CF is associated with systolic LV dilatation, LVH and QT prolongation. Furthermore, in keeping with the impaired cardiomyocyte stress response demonstrated in vitro, we show that acute respiratory exacerbations of CF are associated with a fall in LVEF (also see below).

It has been hypothesised that CF is associated with myocardial fibrosis [27], but other than autopsy reports of myocardial necrosis and fibrosis following infant sudden death, there is little available evidence [28]. MRI-derived ECV quantifies myocardial extracellular matrix volume and in non-acute situations is a robust measure of myocardial fibrosis, having been extensively validated histologically [10]. Myocardial T1 is a surrogate of ECV, however, the values are more scanner- and sequence dependent compared to ECV. Myocardial LGE provides a robust identification of focal replacement fibrosis [9]. Using these techniques we show, for the first time, that stable CF is associated with diffuse interstitial and replacement fibrosis. Indeed, we demonstrate that the identified LVH in CF is predominantly driven by myocardial fibrosis rather than cellular hypertrophy. Hypothesised causes of myocardial fibrosis in CF include recurrent inflammation (see below), chronic hypoxia, activation of the renin–angiotensin–aldosterone system and hyperglycaemia [27]. The impaired LV diastolic function and strain observed in some echocardiographic studies may be secondary to fibrosis [4, 5]. Myocardial fibrosis is consistently associated with adverse outcome in other conditions [29] and investigation of the relationship between myocardial fibrosis and prognosis in CF is required.

In addition to chronic myocardial manifestations, this study demonstrates that acute respiratory CF exacerbation is associated with acute myocardial contractile dysfunction. There was a significant fall in LVEF seen during an acute exacerbation, which normalised in the chronic period. Myocardial ECV and T1 are also reflective of myocardial water content and, in the appropriate clinical context, are markers of myocardial oedema. ECV, T1, and K^trans^ were all higher in acute CF compared to stable CF, although the differences were not statistically significant. This is likely as a result of the small sample size. T2 values were no different between acute and chronic CF. Although T2 was shown to be highly specific for detecting myocardial oedema, it has a lower sensitivity and lower diagnostic accuracy compared to T1 mapping [30, 31] which could explain why T1 mapping detected changes that T2 mapping did not. Despite the lack of statistical significance, the behaviour of ECV, T1 and K^trans^ indices in acute and chronic CF suggests a presence of myocardial oedema at the time of pulmonary exacerbation which then possibly resolves in the chronic period. This finding provides a strong foundation for future, larger studies, appropriately powered to detect the changes in MRI-derived oedema indices. The myocardial manifestations were observed despite the acute MRI scan taking place 12 days post-admission therefore findings should be more apparent if scanning takes place earlier.

Putting the findings together, we hypothesise that an acute respiratory CF exacerbation leads to systemic inflammation, as part of which there is increased myocardial capillary permeability, leading to myocardial oedema. As a result of the oedema, and the aforementioned impaired contractile response of cardiomyocytes to stress, LV function acutely declines. Recurrent episodes of myocardial inflammation may contribute to fibrosis. Larger studies involving histological validation will be required to confirm this hypothesis.

It is important to acknowledge alternative and confounding mechanisms that could contribute to contractile dysfunction in CF patients. Increased large artery stiffness is present in CF patients and is weakly correlated to inflammatory status [32]. Increased afterload during an acute exacerbation could contribute to an impairment of systolic function. CF patients were also shown to have reduced nitric oxide bioavailability and endothelial dysfunction [33]. Although premature coronary artery disease in major epicardial vessels is not usually part of CF disease spectrum [34] and there was no evidence of previous myocardial infarction in any of our participants, coronary microvascular dysfunction was shown to contribute to contractile dysfunction in other conditions [35]. Additionally, hypoxia accompanying an acute CF exacerbation could further worsen endothelial dysfunction [36]. Coronary assessment was not part of the study (see “Limitations” section). Finally, LV contractility can be affected by chronic activation of (CaMKII) and Ca^2+^-activated chloride channels, especially in the presence of increased β-adrenergic stimulation as described above [24].

CF was associated with impaired pulmonary tissue blood flow, which was most marked in the upper lobes, in keeping with the known pattern of disease progression [37]. Pulmonary tissue blood flow increased during acute exacerbation in comparison to stable CF, which may reflect relative hyperaemia as part of the inflammatory response, but the difference was not significant, and it remained substantially lower than in controls. In keeping with previous studies pulmonary T1 was reduced, likely reflecting parenchymal loss, the severity of which correlated strongly with FEV1% [13, 37]. Capillary permeability and pulmonary extracellular volume measurements are dependent on GBCA delivery and mixing, thus were likely underestimated as a result of low pulmonary tissue perfusion.

The severity of the myocardial injury, specifically myocardial oedema, showed a strong association with airflow limitation. This is a key finding and demonstrates the relationship between heart and lung pathophysiology. FEV_1_ is perceived to be the strongest prognostic indicator in CF because it indicates the severity of lung disease, however, it may be that in addition, FEV_1_ is a marker of the severity of myocardial injury. Further investigation is required.

There were no associations between other heart and lung measurements. Indeed, despite improvements in symptoms, spirometry and circulating inflammatory markers between the acute and stable assessments, there were no significant improvements in lung MRI indices. This may indicate that the lung disease in this cohort was at an advanced, essentially irreversible stage, whereas the myocardium was relatively less affected and thus acute-on-chronic injury was evident. Application of the protocol in patients with less severe lung disease would be interesting. The lack of association between heart and lung disease suggests myocardial disease may occur independently of pulmonary disease and indicates the requirement for dedicated cardiac evaluation.

## Limitations

The number of patients included was small, but despite this the results were consistent with preclinical findings and achieved statistical significance. The impact of acute respiratory exacerbation on myocardium may be underestimated as the requirement for patients to hold their breath during many of the MRI acquisitions made it impossible to perform the acute MRI closer to the admission date. Whilst the relationship between ECV and native T1 and histological myocardial fibrosis has been demonstrated in multiple previous studies, histological validation was not performed in the current study, and the relationship between ECV and T1 mapping and histological myocardial fibrosis demonstrated in the previous studies may not necessarily extrapolate to the current study [10, 18]. Anatomical or functional coronary imaging was not part of the study. Although premature coronary artery disease is not usually considered part of CF spectrum and none of the patients had evidence of myocardial infarction on LGE imaging, undiagnosed coronary artery disease could contribute to contractile dysfunction.

## Conclusions

Cardiopulmonary MRI technology allows for simultaneous interrogation of cardiac and pulmonary structure and function, and evaluates their relationship. We demonstrate that stable CF is associated with adverse myocardial remodelling, including systolic LV dilatation and LV hypertrophy, driven by focal replacement and diffuse interstitial myocardial fibrosis. In addition, we demonstrate that acute respiratory exacerbation of CF is associated with acute myocardial contractile dysfunction. There is also a suggestion of myocardial oedema in the acute period, the severity of which is related to pulmonary dysfunction.

### Supplementary Information

Below is the link to the electronic supplementary material.

Supplementary file3 (DOCX 56 kb)

Supplementary file1 (JPG 113 kb)

Supplementary file2 (JPG 99 kb)

## Data Availability

The datasets generated and/or analysed during the current study are available from the corresponding author on reasonable request.

## Abbreviations

CF: Cystic fibrosis
CFTR: Cystic fibrosis transmembrane conductance regulator
DCE Imaging: Dynamic contrast enhanced imaging
ECV: Extracellular volume
EF: Ejection fraction
FEV_1_: Forced expiratory volume in one second
GBCA: Gadolinium based contrast agent
hsTnI: High sensitivity troponin I
K^trans^: Transfer constant (capillary permeability)
LGE: Late gadolinium enhancement
LV: Left ventricle
MRI: Magnetic resonance imaging
