# Supplementary material for: Cardiac involvement in cystic fibrosis evaluated using cardiopulmonary magnetic resonance
Source: Int J Cardiovasc Imaging. 2022 Jan 7;38(5):1121–31. doi: 10.1007/s10554-021-02496-6 (PMC9116982; doi:10.1007/s10554-021-02496-6)
Supplement: Supplementary file 1 — Supplementary file3 (DOCX 56 kb) [file 10554_2021_2496_MOESM1_ESM.docx]

**Cardiac involvement in cystic fibrosis evaluated using cardiopulmonary magnetic resonance**

**Supplementary Material**

**Combined cardiopulmonary magnetic resonance protocol**

The magnetic resonance imaging (MRI) protocol was previously described in detail (1). It included the following sequences (duration 60 minutes; Supplementary Figure 1):

1. Cardiac (long- and short-axis) steady-state free precession (SSFP) cines to measure myocardial function.

2. Basal and mid left ventricular (LV) short axis T1 mapping (MOdified Look-Locker Inversion Recovery (MOLLI)) before and 15 minutes following the final bolus of gadolinium-based contrast agent (GBCA) (see below) for evaluation of myocardial oedema and fibrosis (2). T2 mapping (T2-prepared SSFP) at the same positions to provide further oedema assessment (2). Late gadolinium enhancement (LGE) imaging to assess for focal replacement fibrosis (3, 4).

3. Pulmonary T1 mapping (MOLLI; central sagittal planes) for evaluation of pulmonary injury (5).

4. Dynamic contrast enhanced (DCE) imaging using free-breathing 2D saturation-recovery fast low-angle shot dynamic acquisitions in four planes (basal and mid LV, left and right lung central sagittal planes) to assess myocardial and pulmonary capillary permeability (transfer constant (K^trans^)), pulmonary extracellular volume fraction (Ve) and blood flow (F) (6, 7). GBCA (gadoterate meglumine (Dotarem), Guerbet) was divided into three doses to avoid T2 shortening effects and saturation of the arterial input function (AIF) (8): (i) 0.005mmol/kg low dose bolus followed by 2 minutes of dynamic acquisition to calculate the AIF first-pass peak; (ii) 0.05mmol/kg higher dose bolus followed by 6 minutes of dynamic acquisition to provide higher contrast-to-noise ratio for the AIF and tissue curves’ tails. The low dose curve was multiplied by 10 and combined with the higher dose curve to produce the final AIFs for kinetic analysis (Supplementary Figure 2); (iii) 0.1mmol/kg administered after the dynamic acquisition for LGE imaging and post-contrast T1 mapping.

**MRI analysis**

*Myocardial function:* Cardiac analysis was performed using Circle CVI42 (Circle Cardiovascular Imaging) according to guidelines (9).

*Myocardial and lung tissue characterisation:* T1 and T2 maps were generated in Siemens Argus (Siemens) and transferred into Horos where epicardial, endocardial and blood pool ROIs were drawn. Blood partial volume effects were minimised by using the middle third of myocardium. Lung borders were contoured in Horos (v2.2.0 Horos Project) to define lung regions of interest (ROI) as previously described (5). Myocardial ECV was calculated as described previously (10). LV extracellular matrix mass (g) was calculated by multiplying LV mass by ECV (2). LV cellular mass (g) was calculated by multiplying LV mass by (100%-ECV).

*Myocardial and lung blood flow characteristics:* DCE imaging was analysed using a custom written Matlab code (v9.0, MathWorks). Cardiac motion correction was achieved using an intensity-based rigid registration algorithm. Lung registration was achieved using the Advanced Normalisation Tools symmetric normalization non-linear registration algorithm, employing cross-correlation as the similarity measure (11). Myocardial and lung ROIs were drawn as described above. AIFs were derived from right ventricular (RV) blood pool for pulmonary analysis and LV blood pool for myocardial analysis. Contrast agent kinetics were modelled using an extended Kety model on a voxel-by-voxel basis within registered ROIs to calculate K^trans^ and Ve (6). Pulmonary perfusion was deconvoluted from first pass dynamic data as described previously (6, 7).

**Results**

**Participants characteristics**

CF patients admitted with pulmonary exacerbation received the following antibiotic therapies: tobramycin (n=7), meropenem (n=4), piperacillin / tazobactam (n=2), trimethoprim / sulfamethoxazole (n=2), ceftazidime (n=2), ciprofloxacin (n=1).

**References:**

1. Lagan J, Naish JH, Fortune C, Bradley J, Clark D, Niven R, et al. Myocardial involvement in eosinophilic granulomatosis with polyangiitis evaluated with cardiopulmonary magnetic resonance. Int J Cardiovasc Imaging. 2021;37(4):1371-81.

2. Messroghli DR, Moon JC, Ferreira VM, Grosse-Wortmann L, He T, Kellman P, et al. Clinical recommendations for cardiovascular magnetic resonance mapping of T1, T2, T2* and extracellular volume: A consensus statement by the Society for Cardiovascular Magnetic Resonance (SCMR) endorsed by the European Association for Cardiovascular Imaging (EACVI). J Cardiovasc Magn Reson. 2017;19(1):75.

3. Gulati A, Jabbour A, Ismail TF, Guha K, Khwaja J, Raza S, et al. Association of fibrosis with mortality and sudden cardiac death in patients with nonischemic dilated cardiomyopathy. JAMA. 2013;309(9):896-908.

4. Moravsky G, Ofek E, Rakowski H, Butany J, Williams L, Ralph-Edwards A, et al. Myocardial fibrosis in hypertrophic cardiomyopathy: accurate reflection of histopathological findings by CMR. JACC Cardiovasc Imaging. 2013;6(5):587-96.

5. Donnola SB, Dasenbrook EC, Weaver D, Lu L, Gupta K, Prabhakaran A, et al. Preliminary comparison of normalized T1 and non-contrast perfusion MRI assessments of regional lung disease in cystic fibrosis patients. J Cyst Fibros. 2017;16(2):283-90.

6. Naish JH, Kershaw LE, Buckley DL, Jackson A, Waterton JC, Parker GJM. Modeling of contrast agent kinetics in the lung using T1-weighted dynamic contrast-enhanced MRI. Magnetic resonance in medicine. 2009;61(6):1507-14.

7. Hueper K, Parikh MA, Prince MR, Schoenfeld C, Liu C, Bluemke DA, et al. Quantitative and semiquantitative measures of regional pulmonary microvascular perfusion by magnetic resonance imaging and their relationships to global lung perfusion and lung diffusing capacity: the multiethnic study of atherosclerosis chronic obstructive pulmonary disease study. Invest Radiol. 2013;48(4):223-30.

8. Roberts TP. Physiologic measurements by contrast-enhanced MR imaging: expectations and limitations. Journal of magnetic resonance imaging : JMRI. 1997;7(1):82-90.

9. Schulz-Menger J, Bluemke DA, Bremerich J, Flamm SD, Fogel MA, Friedrich MG, et al. Standardized image interpretation and post processing in cardiovascular magnetic resonance: Society for Cardiovascular Magnetic Resonance (SCMR) board of trustees task force on standardized post processing. J Cardiovasc Magn Reson. 2013;15(1):35.

10. Miller CA, Naish JH, Bishop P, Coutts G, Clark D, Zhao S, et al. Comprehensive validation of cardiovascular magnetic resonance techniques for the assessment of myocardial extracellular volume. Circ Cardiovasc Imaging. 2013;6(3):373-83.

11. Avants BB, Epstein CL, Grossman M, Gee JC. Symmetric diffeomorphic image registration with cross-correlation: evaluating automated labeling of elderly and neurodegenerative brain. Medical image analysis. 2008;12(1):26-41.

**Supplementary Figures Legends**

**Supplementary Figure 1. Cardiopulmonary magnetic resonance imaging protocol.** DCE – dynamic contrast enhanced; GBCA – gadolinium based contrast agent; HASTE - Half-Fourier-Acquired Single-shot Turbo spin Echo; LGE – Late enhancement imaging.

**Supplementary Figure 2.** **Example right ventricular arterial input function (RV AIF).** (a) Low dose (x10; non-bold line) and high dose (bold line) RV concentration curves. Significant underestimation of the peak concentration is evident in the high dose curve during the first pass peak. Beyond the peak, the curves are seen to align closely. (b) Resulting composite RV AIF.
