# Supplementary figures and images for: Cardiac involvement in cystic fibrosis evaluated using cardiopulmonary magnetic resonance
Source: Int J Cardiovasc Imaging. 2022 Jan 7;38(5):1121–31. doi: 10.1007/s10554-021-02496-6 (PMC9116982; doi:10.1007/s10554-021-02496-6)

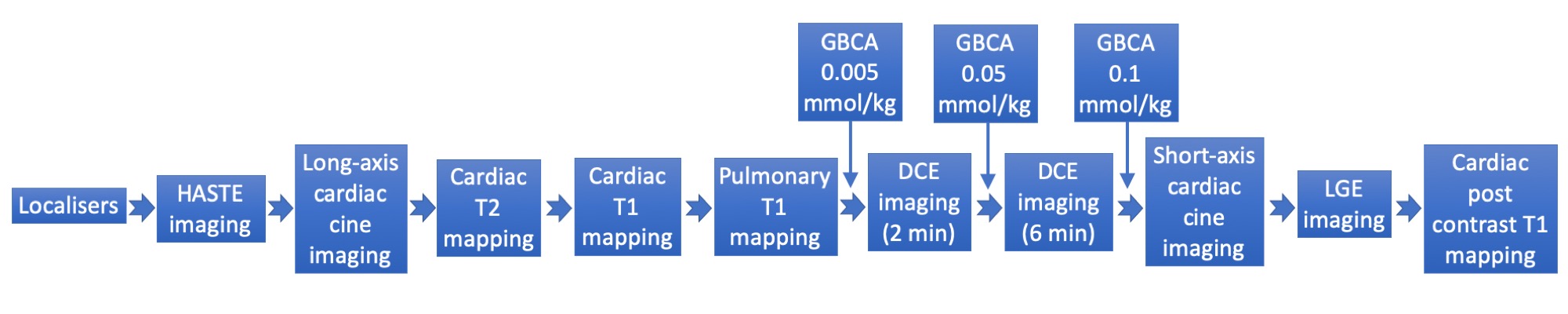

Supplement: Supplementary file 2 — Supplementary file1 (JPG 113 kb) [file 10554_2021_2496_MOESM2_ESM.jpg]

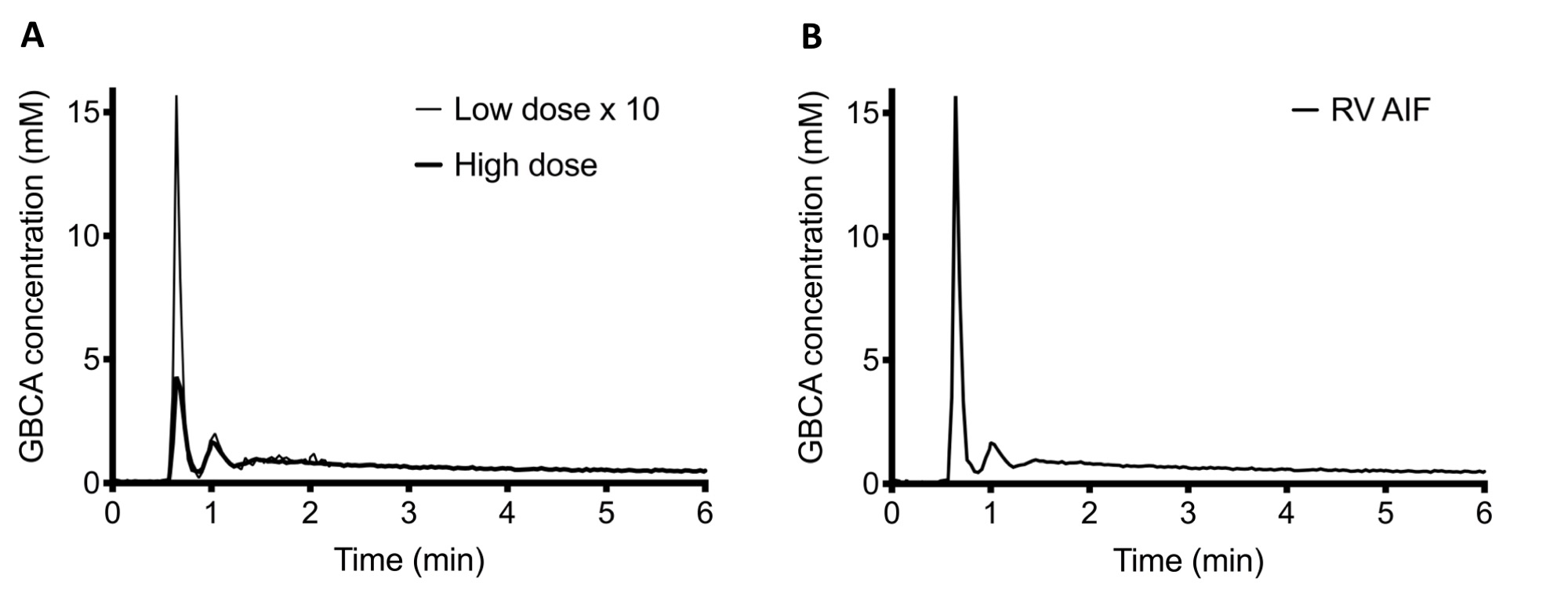

Supplement: Supplementary file 3 — Supplementary file2 (JPG 99 kb) [file 10554_2021_2496_MOESM3_ESM.jpg]
